# Calculation of the 3-D femoral component’s orientation in total hip arthroplasty using a trigonometric algorithm

**DOI:** 10.1038/s41598-022-07331-z

**Published:** 2022-03-03

**Authors:** Joost H. J. van Erp, Tom P. C. Schlösser, Ariënne W. Baijense, Thom E. Snijders, Rob Stevenson, Willem Paul Gielis, René M. Castelein, Harrie Weinans, Arthur de Gast

**Affiliations:** 1 Clinical Orthopedic Research Center-mN, Zeist, The Netherlands; 2 Department of Orthopedic Surgery, Diakonessenhuis, Utrecht, The Netherlands; 3 Department of Orthopedic Surgery, University Medical Center Utrecht, Utrecht, The Netherlands; 4 Korteweg-de Vries Institute for Mathematics, University of Amsterdam, Amsterdam, The Netherlands; 5 Department of Biomechanical Engineering, TU Delft, Delft, The Netherlands

**Keywords:** Biophysics, Biotechnology, Anatomy, Signs and symptoms, Mathematics and computing

## Abstract

Femoral component orientation plays a key role in implant stability and therefore the success rate of total hip arthroplasty. To date, this topic has been studied using various definitions and a variety of imaging modalities and protocols. The aim of this study is a proof of concept that a new algorithm can be used to describe the femoral component’s 3D orientation on the three orthogonal anatomical planes and relative to its mechanical axis using input from two orthogonal planes. CT scans of 18 patients with a total of 22 hip arthroplasties were collected. From these, orthogonal coronal and sagittal projections of the complete femur were acquired in the scanning position (MIPs) and relative to the femoral mechanical axis (corrected MIPs). On these images, the orientation of the neck of the femoral component in space and relative to the femoral axis, respectively, was measured by coronal inclination (CI_F_), sagittal inclination (SI_F_) and transverse version (TV_F_). With the algorithm, TV_F_ was also calculated based on CI_F_ and SI_F_. Differences between measured and calculated TV_F_ and intra- and inter-observer reliability were evaluated using intra-class correlation coefficients (ICC). The error of non-orthogonal imaging (85° angle between the sagittal and coronal reconstructions) was tested on a third series of MIPs. The ICC between the calculated TV_F_ and manually measured TV_F_, in space and relative to the femoral axis, was 0.98 for both with median absolute differences of 1.3 and 1.5°. For non-orthogonal images this was 0.70 with a median absolute difference of 5°. ICCs for intra-observer and inter-observer reliability for the calculated TV_F_ values were 0.98 and 0.88, respectively. With this algorithm the transverse orientation of the neck of the femoral component can be assessed in space and relative to the mechanical femoral axis by combining its sagittal and coronal orientation. As long as the imaging visualizes two orthogonal planes, the orientation of an implant can be assessed in 3-D, regardless of the imaging modality.

## Introduction

Total hip arthroplasty (THA) has become one of the most successful surgeries of the last fifty years being very effective in relieving pain and improving hip function^1^. The number of THAs has increased over the last decades and is still increasing due to aging of the population and higher demand and activity level^2^. Yet THA can lead to challenging complications, such as dislocation^3^. The risk of dislocation after primary THA for osteoarthritis (OA) has been reported to be 0.3–10% in systematic reviews^4,5^. The orientation of the acetabular component has a proven impact on the stability of THA and has been widely studied^6,7^. Femoral component orientation, however, plays a key role in implant stability and success rate of total hip arthroplasty as well^8–11^ .

The orientation of a THA has been described using various definitions and conflicting terminology on 2-D and 3-D radiographic images, acquired in different body positions^12–14^. In order to avoid more confusion on this topic, in 2019, the Hip-Spine Workgroup published a consensus review in which terminology was standardized to systematically approach the role of implant positioning and the hip-spine relation for the acetabular component^12^. Terminology for description of the 3-D orientation of the femoral component, however, was not included. In order for hip surgeons to easily assess the 3-D orientation of the neck of the femoral component on various 2-D and 3-D images we introduce a simple trigonometric algorithm. Application of this trigonometric algorithm to calculate the transverse plane orientation, using the sagittal and coronal orientation has previously been validated for the acetabular component^15,16^. This algorithm can also be used to describe the mathematical relation between the femoral component orientation on the three orthogonal anatomical planes, in space and in relation to the mechanical axis of the femur. Therefore, the aim of this study is a proof of concept that this algorithm can be used to describe the femoral component`s 3D orientation in space and relative to its mechanical axis using input from two orthogonal planes.

## Materials and methods

### Study population

CT images including the pelvis and complete femur that were acquired for analysis of vascular pathology and had a THA in situ, were systematically retrieved from the patient archiving system after approval from the Institutional Review Board. The need for ethics approval and informed consent was waived by the Institutional Review Board, according to national legislation (Study reference number 17.037). Clinical and radiographic charts were reviewed for in- and exclusion and collection of demographical data. Exclusion criteria were previous ipsilateral hip surgery other than primary THA, metastatic disease localized in the pelvis or femur and image series that were incomplete or with substantial artifacts. All scans were acquired in supine position using a 16-channel multidetector CT system (Siemens Healthcare, Erlangen, Germany) between March 2013 and October 2016. After inclusion scans were fully anonymized.

### Image analyses

A series of multiplanar reconstructions of the coronal, transverse and sagittal plane were automatically generated in Sectra (Sectra AB, Linköping, Sweden) using three-dimensional maximum intensity projection (MIP, slice thickness 50 mm) centered on both femoral heads. Coronal and sagittal MIPs were used to resemble anterior–posterior (AP) and lateral femoral radiographs, because MIP volume rendering is defined as a projection of the voxels with maximum intensity that fall in the way of parallel rays traced from the viewpoint to the plane of projection^17^. To correct for scanning position and taking account the orientation of the femur, the posterior condylar plane and the mechanical femoral axis were manually identified and used for reconstruction of a second series of 3-D MIPs (corrected MIPs). The posterior condylar plane was identified in the transverse plane and the mechanical femoral axis was defined as the line connecting the center of the knee joint and the center of the femoral head in the coronal and sagittal plane. On MIPs in the scanning position and corrected-MIPs, angular parameters were manually measured to define the 3-D orientation of the neck of the femoral component in space and relative to the femoral mechanical axis, respectively. To study the effect of non-orthogonal radiographic imaging and mimic daily clinical practice, a third series with ‘out-of-plane’ MIPs was reconstructed with a 85° angle between the sagittal and coronal reconstructions.

**Figure 1:**
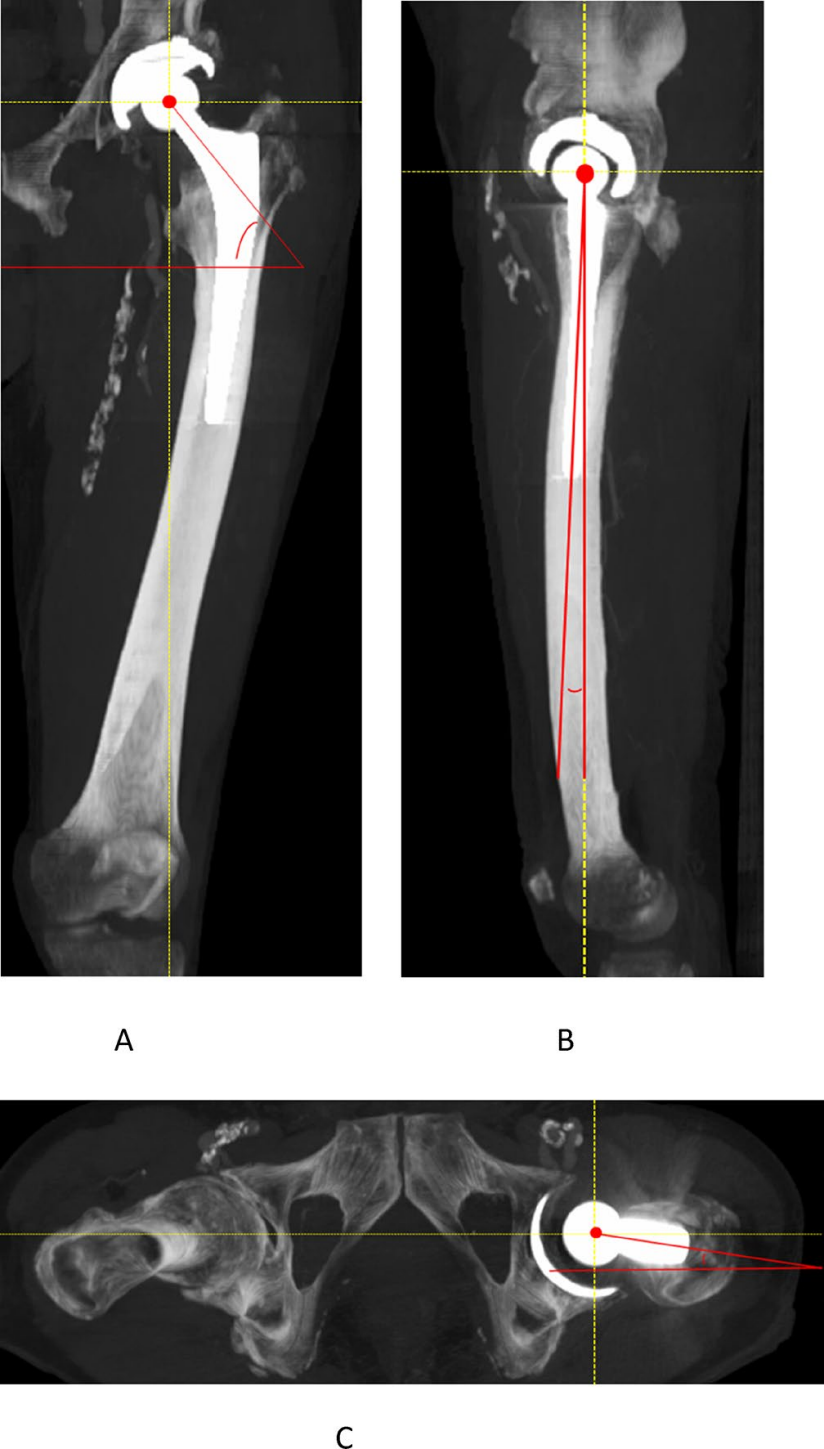
Measurement method of coronal inclination (CI_F_, defined as the angle between the line through the longitudinal axis of the neck of the femoral component and the horizontal) (**A**), sagittal inclination (SI_F_, defined as the angle between the line from the center of the femoral head to through the middle of the femoral neck, in relation to the vertical) (**B**) and transverse version (TV_F_, defined as the angle from the center of the femoral head through the femoral neck, in relation to the horizontal (**C**) of the femoral component on 3-D MIP constructed CT (horizontal/vertical displayed as dashed line).

**Figure 2:**
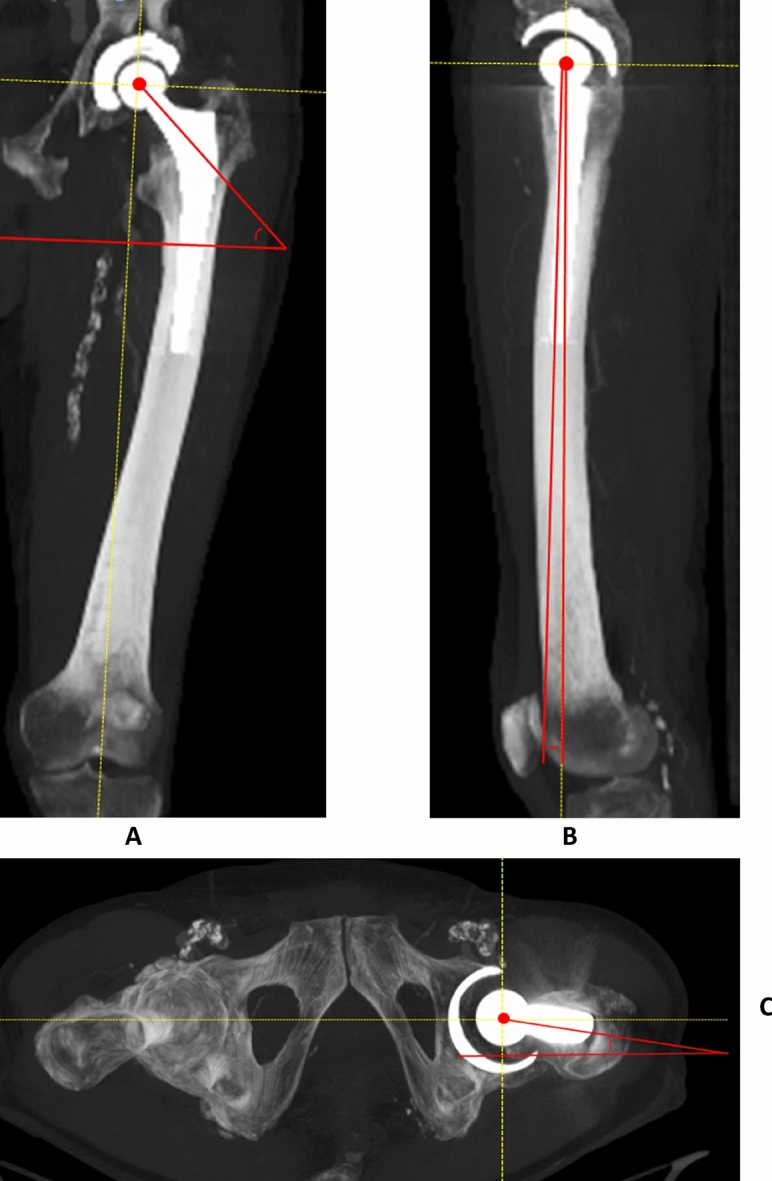
Measurement method of coronal inclination (CI_F’_, defined as the angle between the line through the longitudinal axis of the neck of the femoral component and the horizontal on the mechanical femoral axis) (**a**), sagittal inclination (SI_F’_ defined as the angle between the line from the center of the femoral head to through the middle of the femoral neck, in relation to the mechanical femoral axis) (**b**) and transverse version (TV_F’_, defined as the angle from the center of the femoral head through the femoral neck, in relation to the horizontal on the mechanical femoral axis, with the posterior condylar plane as distal reference) (**c**) of the femoral component on 3-D MIP constructed CT (mechanical femoral axis/horizontal displayed as dashed line).

Similar to the definitions for description of the acetabular component orientation as outlined by Hip-Spine Workgroup the following, definitions were used to describe the 3-D orientation of the neck of the femoral component^12^:

- Coronal femoral inclination in space (CI_F_) and relative to the mechanical femoral axis (CI_F’_) were defined as the angle between the line through the longitudinal axis of the neck of the femoral component and the horizontal on the MIPs and corrected MIPs, respectively (Figs. 1 and 2). Varus alignment corresponded with low CI_F_, and valgus with high CI_F_.
- The sagittal inclination (SI_F_) and mechanical SI_F’_ were measured on the sagittal images as the angle between the line from the center of the femoral head through the center of the femoral neck, in relation to the vertical, on the MIPs and corrected MIPs, respectively (Figs. 1 and 2). Ante-inclination of the femoral component relative to the femur corresponded with high SI_F_/SI_F’_.
- Transverse version (TV_F_) and mechanical TV_F’_ were measured on the transverse images and were defined as the angle between the line from the center of the femoral head through the center of the femoral neck in relation to the horizontal. For the second series of MIPs, TV_F’_ was measured with the posterior condylar plane as distal reference MIPs (Figs. 1 and 2). Anteversion of the femoral component corresponded with higher TV_F_/TV_F’_.

For intra-observer reliability, one observer measured the orientation parameters four times in separate sittings. For inter-observer reliability, a second observer performed the same measurements. Since this project aims to calculate the transverse plane implant orientation based on sagittal and coronal data, all these measurements were performed for TV_F_ only_,_ the orientation parameter most difficult to assess on conventional radiographs.

### Trigonometric algorithm

The trigonometric algorithm combines the mathematical relation of orientation parameters in two orthogonal planes to calculate the parameter in the third orthogonal plane^18^. The following base equations were used:

$${\mathrm{CI}}_{\mathrm{F}}=\mathrm{arctan}\left(\frac{\mathrm{tan}\left({\mathrm{TV}}_{\mathrm{F}}\right)}{\mathrm{tan}\left({\mathrm{SI}}_{\mathrm{F}}\right)}\right)$$

$${\mathrm{SI}}_{\mathrm{F}}=\mathrm{arctan}\left(\frac{\mathrm{tan}\left({\mathrm{TV}}_{\mathrm{F}}\right)}{\mathrm{tan}\left({\mathrm{CI}}_{\mathrm{F}}\right)}\right)$$

$${\mathrm{TV}}_{\mathrm{F}}=\mathrm{arctan}\left(\mathrm{tan}\left({\mathrm{SI}}_{\mathrm{F}}\right)\times \mathrm{tan}\left({\mathrm{CI}}_{\mathrm{F}}\right)\right)$$

Using this algorithm, TV_F_ and TV_F’_ were calculated using the CI_F_/CI_F’_ and SI_F_/SI_F’_ on the coronal and sagittal MIPs and compared to the manual measurement of the TV_F_ and TV_F’_ on CT-scans.

### Statistical analysis

The measured parameters and the equations were imported in Microsoft Excel 2010 (Microsoft Corporation, Redmond, Washington). Statistical analyses were performed using IBM-SPSS Statistics 25 (SPSS Inc., Chicago, Illinois). Continuous parameters were assessed for normality of distribution and shown as mean ± standard deviation (SD) (range). Bland–Altman plots including 95% limit of agreements were produced to compare manual and calculated orientation angles to identity potential systematic errors^19^. For validity analyses, differences between the manual and calculated transverse orientation angles were assessed using the two-way mixed ICC for absolute agreement and corresponding 95%-confidence interval (CI). Additionally, median absolute differences were calculated. For assessment of intra- and inter-observer reliability, calculated parameters were compared within and between the observers using the two-way random ICCs for absolute agreement for intra-observers and the two-way mixed effects model for inter-observers.

### Ethics approval

This study was approved by the local institutional review board.

### Consent to participate

All authors agreed to participate.

### Consent for publication

All authors are in agreement with the manuscript.

## Results

### Demographics

22 THAs, in 18 patients met the inclusion criteria. Demographics are shown in Table 1. Patients were 78 ± 9 (62–90) years of age. Primary THAs were implanted between 1994 and 2016. Three patients were included with a revised THA. Of the femoral components, thirteen (59%) were an uncemented Twinsys stem (Mathys Ltd. Bettlach, Switzerland), three a cemented CLS steel (CLS Spotorno, Zimmer Ltd, Warsaw, United States of America), two an uncemented Richards TI-FIT (Smith & Nephew, Memphis, United States of America), two a cemented Spectron (Smith & Nephew, Memphis, United States of America), and two an uncemented Euroform (DePuy Synthes, DePuy Ortopaedics, Warsaw, United States of America).

**Table 1:** Demographics.

Total n = 18 patients	
Females, n	12 (67%)
THA	22
Age in years, mean ± SD	78 ± 9
Uncemented stem, n	16 (73%)
Head size 28 mm	8 (36%)
Head size 32 mm	12 (55%)
Head size unknown	2 (9%)

### Manual measurements vs calculated measurements

**Figure 3:**
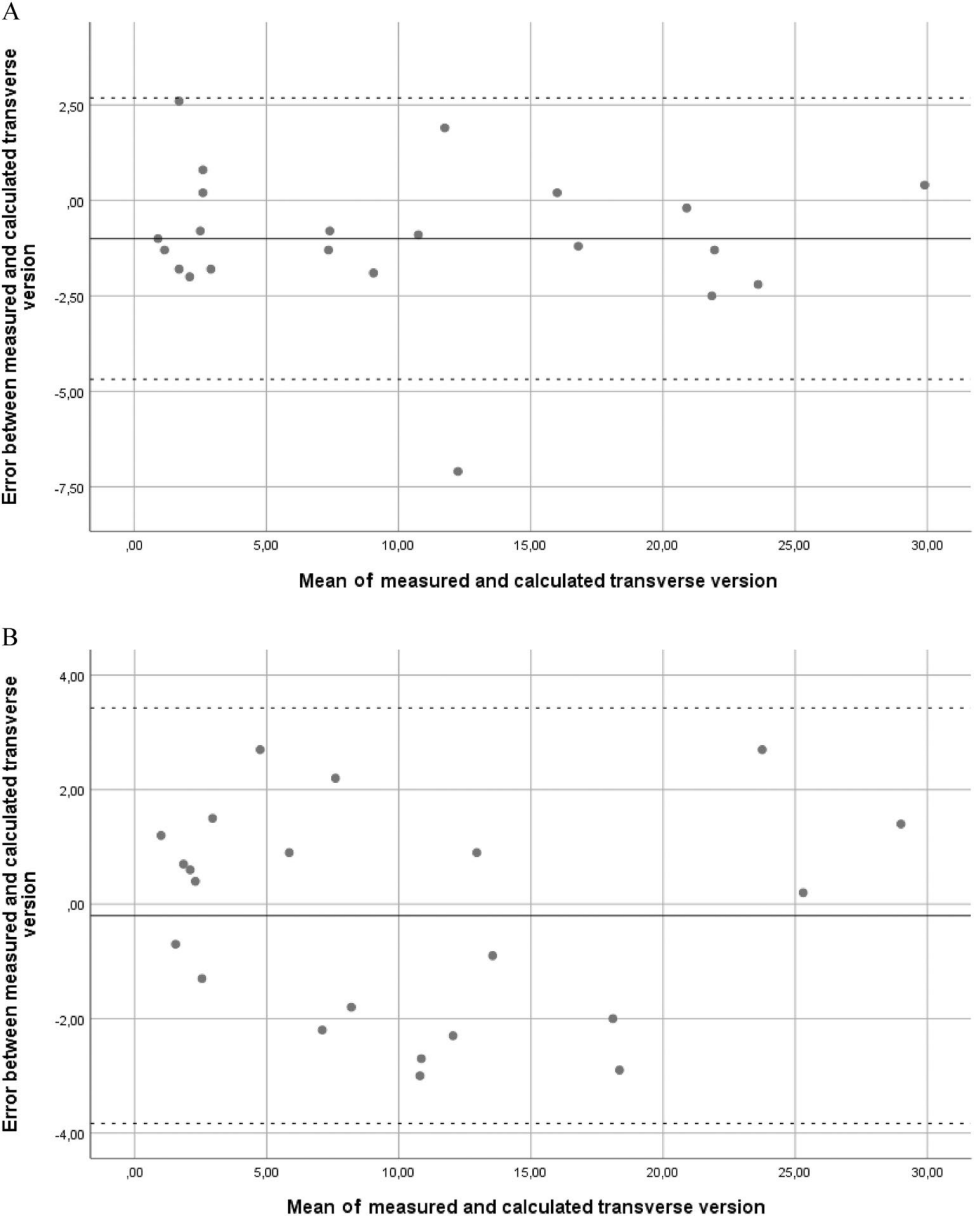
Bland–Altman plots showing the error between manual and calculated measurements. The full line depicts the mean error, the dotted line indicates the 95% confidence interval. (**A**) Transverse version uncorrected for mechanical angle. (**B**) Transverse version corrected for mechanical angle.

The manually measured orientation parameters were normally or lognormally distributed, implying ICC could be used in further analysis^20^. CI_F_, SI_F_ and TV_F_, measured on the 3-D MIP reconstructions were 50.0° ± 9° (26.1–72.0), 11.3° ± 9° (1.8–31.6) and 10.8° ± 9° (0.4–29.7), respectively. CI_F’_, SI_F’_ and TV_F’_ were 51.4° ± 4° (43.1–62.6), 12.2° ± 8° (1.8–33.0) and 10.2° ± 8° (0.4–28.3), respectively. The Bland–Altman plots did not show systematic errors between manually measured and calculated values (Fig. 3). The 95% limits of agreement indicate the error between the calculated and measured angle is less than 4° for transverse version in 95% of the cases. The manually measured and calculated TV_F_, and TV_F’_ are shown in Table 2. ICCs between the manual and calculated TV_F_ were 0.98 on both series, and the median absolute differences were less than 2°. To test the validity of using the algorithm on non-orthogonal images, calculated TV_F_ was compared to calculated TV_F_ performed with non-orthogonal images, the ICC was 0.70 and the median absolute difference 5° (Table 3).

**Table 2:** The manually measured and calculated TV_F_ and TV_F’_ are shown in degrees as mean ± SD (range).

	Manual	Calculated	Absolute difference	ICC
TV_F_	10.8° ± 9° (0.4–29.7)	9.8° ± 9° (0.4–30.1)	1.3° (0.8–1.9)	0.98 (0.95–0.99)
TV_F’_	10.2° ± 8° (0.4–28.3)	10.0° ± 8° (1.2–29.7)	1.5° (0.9–2.4)	0.98 (0.94–0.99)

The absolute differences is demonstrated as median {interquartile range} due to a non-normal distribution. The intraclass correlation (ICC) is shown with the 95% confidence interval.

**Table 3:** To test the validity of using the algorithm on non-orthogonal images, calculated TV_F_ was compared to calculated TV_F_ performed with non-orthogonal images using the ICC.

	Calculated TV_F_ on orthogonal images	Calculated TV_F_ on non-orthogonal images	Absolute difference	ICC
TV_F_	9.8° ± 9° (0.4–30.1)	5.7° ± 5.6° (0.4–23.1)	5.0° (1.8–8.4)	0.70 (0.14–0.89)

Measurements are demonstrated as mean ± SD (range). Difference is demonstrated as absolute difference as median {interquartile range} due to a non-normal distribution. The ICC is shown with the 95% confidence interval (CI).

### Intra and inter-observer reliability

ICCs for intra-observer and inter-observer reliability were 0.98 and 0.88, respectively (Tables 4 and 5).

**Table 4:** For intra-observer reliability analyses, four measurements of one observer of calculated TV_F_ were compared using the ICC.

	Measurement 1	Measurement 2	Measurement 3	Measurement 4	ICC (CI)
Calculated TV_F_	10.9° ± 9.6° (0.3–36.2)	10.9° ± 9.8° (0.6–37.1)	10.9° ± 9.8° (0.5–38.1)	11.8° ± 9.4° (0.2–36.5)	0.98 (0.96–0.99)

Measurements are demonstrated as mean ± SD and range. The ICC is shown with the 95% confidence interval (CI).

**Table 5:** For inter-observer reliability analyses, measurements of two observers of calculated TV_F_ were compared using the ICC.

	Observer 1	Observer 2	ICC (CI)
Calculated TV_F_	11.1° ± 9.6° (0.4–37.0)	12.9° ± 10.6° (0.9–35.5)	0.88 (0.74–0.94)

Measurements are demonstrated as mean ± SD and range. The ICC is shown with the 95% confidence interval (CI).

## Discussion

The risk of dislocation after THA is reported to be 0.3%-10% over the last decades, in which malposition of the prosthetic components is considered an important factor^21–23^. The orientation of a THA has been widely assessed on 2-D and 3-D images acquired with different imaging modalities, in different body positions, using a variety of definitions and conflicting terminology^7^. As of yet, no method for assessment of the 3-D orientation of the neck of the femoral component is available for uniform application to different imaging modalities and implant types.

This validation study demonstrates that the transverse orientation of the neck of the femoral component can be accurately assessed by combining the coronal and sagittal orientation of the neck of the femoral component, on for example biplanar radiographs (anterior–posterior and lateral), with a straightforward algorithm. This creates the opportunity for evaluation of femoral component orientation in 3-D, without the need for CT, and in the weight-bearing position. The method described in this proof-of-concept study can be applied to radiographs. Therefore, it could easily provide surgeons accurate feedback on the postoperative 3-D implant orientation and offers potential for comparison of the 3-D implant orientation across studies that used different modalities.

Practically, this method requires perfect orthogonal imaging of the implant and patient, thus traditional axiolateral/ crosslateral radiography of the proximal femur is insufficient^15,16^. Innovative modalities such as biplanar radiography could easily provide such images with acceptable radiation exposure and costs for daily clinical practice. Non-orthogonal images could diminish the accuracy of the method. This could be easily controlled, by verification that both femoral heads overlap completely on the lateral radiograph. Furthermore, semi-automatic image processing to determine the exact center of the femoral head and axis of the neck may further reduce the error. To guarantee the theoretically excellent accuracy of a mathematical model, a strict protocol for image acquisition and processing is necessary in clinical practice. In case of non-orthogonal imaging and manual measurement of the coronal and sagittal orientation, the algorithm is still valid. However, it should be noted that the outcomes of calculated TV_F_ will be less accurate.

The orientation of the neck of the femoral implant was assessed in 3-D, by means of the three components of rotation relative to the scanning position, as well as relative to the mechanical femoral axis. The cranio-caudal, medio-lateral and antero-posterior translations, however, are not included in this assessment, since this is highly variable between individuals and implant types. According to a recent international consensus, the orientation parameters (CI, SI, TV) used in this study are defined in such a way that they are uniform and valid for the orientation of the neck of any type of femoral implant in THA surgery and any imaging modality. These parameters represent the basic 3-D orientation of the proximal part of the femoral implant that connects the proximal femur to the center of rotation of the acetabular cup, potentially very important for assessment of implant stability. Comparison of calculated TV_F_ and TV_F’_ showed only small differences. This can be explained by minimal deviations of the mechanical axis of the femoral orientation to the scanning position and orientation of the scan. The long arm of the coronal and sagittal orientation of the mechanical femoral axis, with minimal impact on the proximal implant orientation is another explanation. Both TV_F_ and TV_F’_ may be relevant in clinical practice, since the first can describe the functional orientation of the femoral implant in space, while the second describes the intrafemoral alignment.

The proposed algorithm can calculate TV_F_ in a valid and reliable way based on CI_F_ and SI_F_, with excellent validity^24^ (Table 2) and excellent and good intra- and inter-observer reliability respectively (Tables 4, 5). The mean absolute difference of our method was < 2° (Table 2), precise enough to enable its use in for the measurement of TV_F_ in daily clinical practice, where TV_F_ usually varies between -10° and 30°. Although Snijders et al. recommended careful use of the algorithm in cases in which two angles are approaching 0°^18^, use of MIP results in more precise measurements, which enables the use the algorithm even when both TV_F_ and SI_F_ are approaching 0°. Type of steel or head size had no impact on the validity of the algorithm, since only the neck-shaft of the prosthesis is used for calculations. The limited sample size of our study is a limitation of this study. The proof of concept of the mathematical algorithm will not change with a larger sample size, however there is a limitation in the measurement of reliability of our method.

The method described provides improvements for the evaluation of optimal femoral component positioning in THA, without a CT. This speeds up the process, allows for easily accessible postoperative feedback, gives better insight and offers great potential for future comparative studies. Additionally, this method could be cost effective and beneficial for patients safety, by reducing radiation exposure. The mean radiation exposure with our two biplane radiographs was 0.8 mSv, while conventional THA CT resulted in a mean of 10.6 mSv. Hence, with our method applied to biplane radiographs, a significant reduction in radiation exposure could be established. While very little is still known about the effect of the femoral component orientation, the described method can be of high relevance for defining the orientation of the femoral component uniformly. With this algorithm it is possible for clinicians to calculate the transverse femoral component alignment in THA patients when they assess implant position on a combination of conventional anterior–posterior and a lateral radiograph including the proximal femur. Furthermore, biplanar radiography techniques such as EOS^TM^ also allow for these analyses. Compared to 3-D imaging techniques, the biplanar radiographs are faster, cheaper part of the clinical routine and enables analysis in the upright position, with significantly lower radiation exposure. The presented method can act as the basis for new research studying the consequences of the femoral component orientation on implant stability.

## Conclusion

The use of the trigonometric algorithm to calculate the 3-D femoral orientation with the newly introduced method is valid and reliable. Transverse femoral component orientation can be simply assessed by combining the sagittal and coronal orientation of the neck of the femoral component on lateral and AP radiographs.
